# Supplementary material for: Fibrin, Bone Marrow Cells and Macrophages Interactively Modulate Cardiomyoblast Fate
Source: Biomedicines. 2022 Feb 23;10(3):527. doi: 10.3390/biomedicines10030527 (PMC8945703; doi:10.3390/biomedicines10030527)
Supplement: Supplementary file 1 [file biomedicines-10-00527-s001.zip › Figures +Table S2.pdf]

Supplementary data

Fibrin, Bone Marrow Cells and macrophages interactively modulate cardiomyoblast fate.

Inês Borrego [1], Aurélien Frobert [1], Guillaume Ajalbert [1], Jérémy Valentin [1], Cyrielle Kaltenrieder[1], Benoît Fellay [3], Michael Stumpe[2], Stéphane Cook [1,3], Joern Dengjel [2], Marie-Noelle Giraud [1] *

Figure S1: CD68+, CD206+ macrophages within the infarcted area and border zone were similar in all groups. Immunostainings for CD206 (A, C) and CD68 (B, D) were performed on paraffin-embedded heart sections. 5 to 6 cross-sections from a systematic sampling of the whole heart were averaged for each animal. Hearts were harvested 4 or 12 weeks post-treatment.

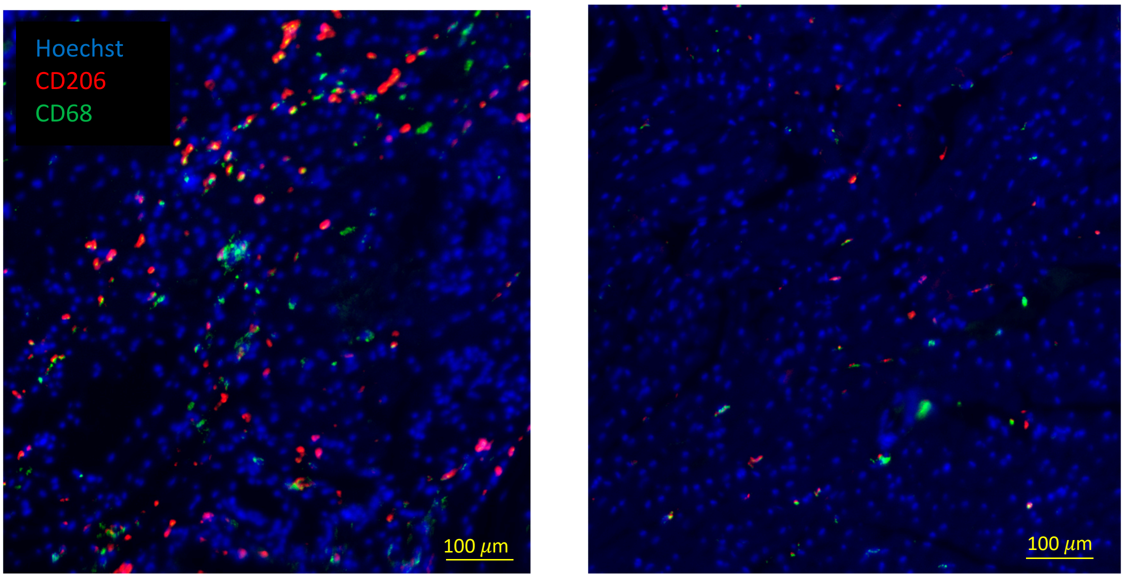

Infarct area

Peri-infarct area

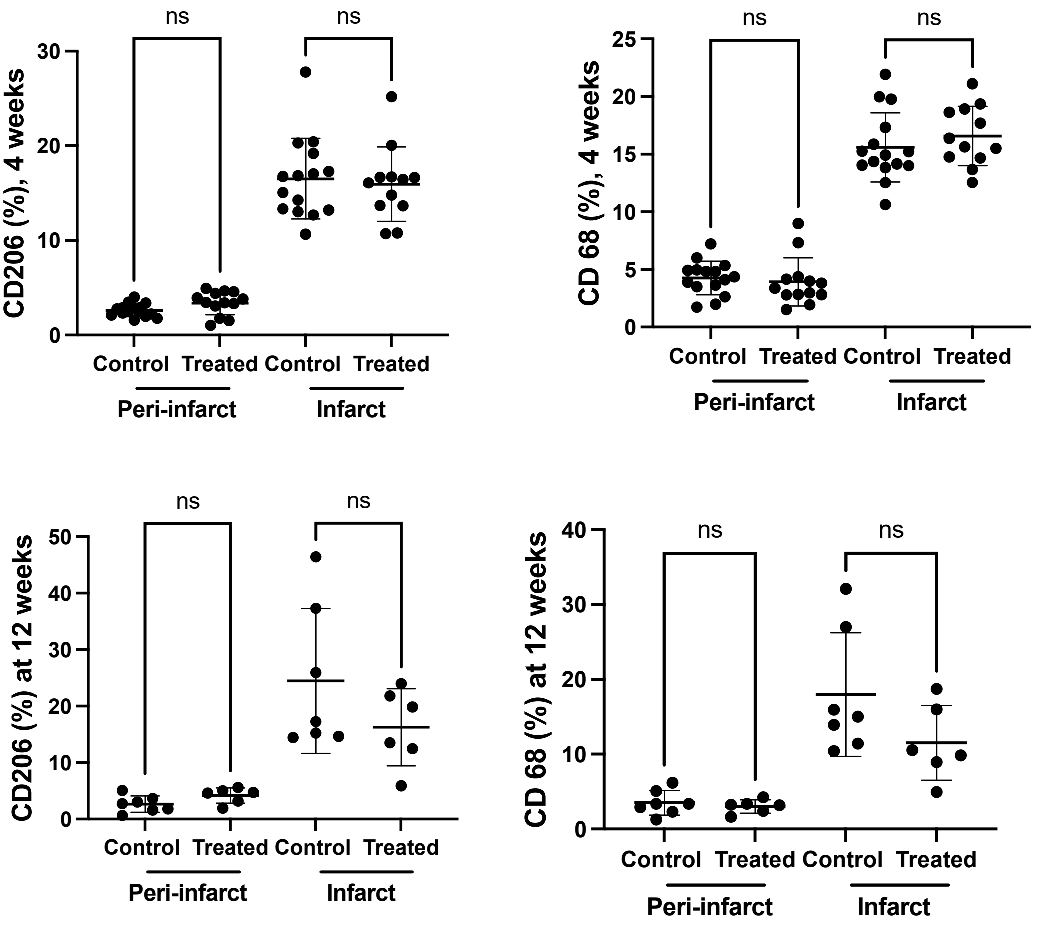
ns
ns
CD206 (%), 4 weeks
30
20
10
0
Control
Treated
Control
Treated
Peri-infarct
Infarct
ns
ns
CD 68 (%), 4 weeks
25
20
15
10
5
0
Control
Treated
Control
Treated
Peri-infarct
Infarct
ns
ns
CD206 (%) at 12 weeks
50
40
30
20
10
0
Control
Treated
Control
Treated
Peri-infarct
Infarct
ns
ns
CD 68 (%) at 12 weeks
40
30
20
10
0
Control
Treated
Control
Treated
Peri-infarct
Infarct

Methods:

Macrophages immunohistochemistry: Samples from rat hearts sectioned into 5-6 parts starting at the base and going to the apex were used for double fluorescent immunochemistry staining. Anti-CD206 (Abcam, ab64693) and anti-CD68 (Abcam, ab31630) were used as primary antibodies and Donkey anti-Rabbit Alexa Fluor® 647 (Abcam, ab150075) and Goat anti-mouse Alexa Fluor® 488 (Abcam, ab150113) were used as secondary antibodies. The tissue sections were counterstained with Hoechst. Images of the left ventricle were acquired using a DM6B bright-field microscope (Leica) at 10x magnification. The quantification was done on FIJJ (ImageJ). Two regions within the infarct zone and the adjacent infarct zone were delimited for each image. An auto-threshold was performed. On each region of interest (ROI), the number of CD206 and CD68 positive cells, Hoechst stained nuclei and the total area covered by these cells were automatically counted. Each image's masks were inspected manually to verify the automatic counting. $CD206^+$ and $CD68^+$ cells ratio was calculated by dividing the number of $CD206^+$ or $CD68^+$ cells by the total nuclei for each ROI.

Figure S2: Surface markers of BMCs measured by flow cytometry and immunostaining. (A) Histogram represents the flow cytometry results, and the pictures represent the immunostainings of the cells. Scale bar represent 100 μm.BMC are a heterogeneous population of cells as indicated by markers from the mesenchymal (CD90, CD29, CD13) and hematopoietic (CD45, CD44, CD68) lineages. The mesenchymal CD90^{+} cells were most abundant. They form a population of large and spread cells. (B) Quantification of the immunostainings

A

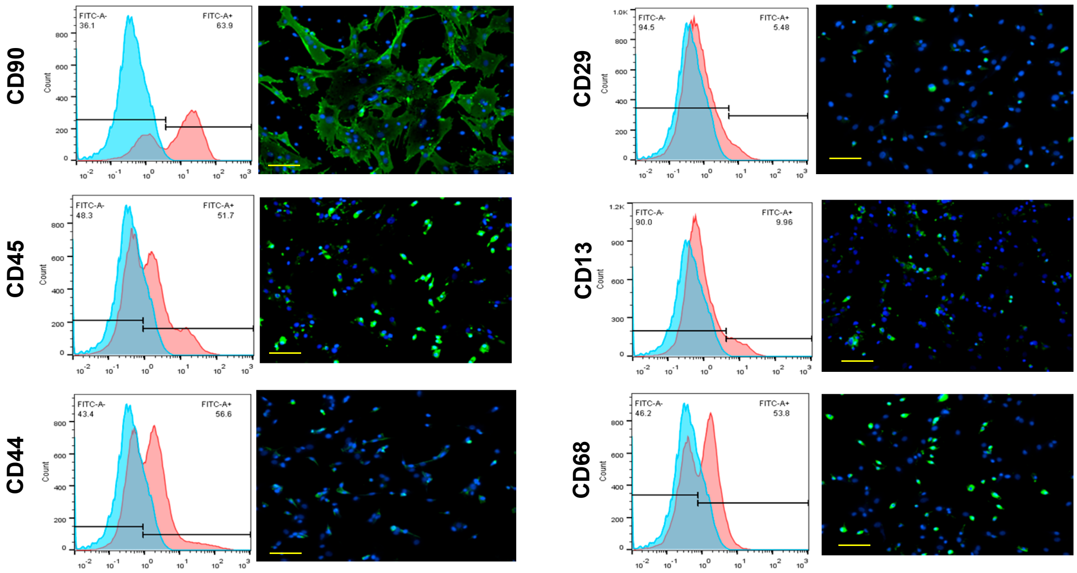

B

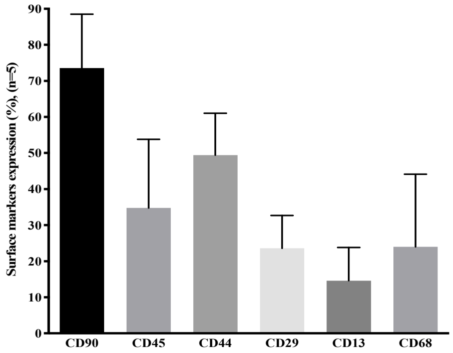

Methods:

Five different pools of BMC (pools of 10 or 3 animals) used in this study were characterised by flow cytometry. Passage 2 cells were detached with accutase. Cell surface markers were analysed using unlabelled Anti-CD90 (rabbit; ab225; Abcam, United Kingdom), anti-CD45 (ab10558, RRID:AB_442810, Abcam, United Kingdom), anti-CD44 (ab119348, RRID:AB_10902529, Abcam, United Kingdom), anti-CD29 (ab179471; RRID:AB_2773020, Abcam, United Kingdom), anti-CD13 (ab108310, RRID:AB_10866195, Abcam, United Kingdom) and anti-CD68 (ab31630, RRID:AB_1141557, Abcam, United Kingdom) in 1:50 dilution. Cells were

further labelled with a secondary antibody (1:500 dilution), including Alexa Fluor 488-conjugated anti-rabbit (ab150077, RRID: AB_2630356, Abcam, United Kingdom), Chromeo 488-conjugated anti-mouse (ab60313, RRID: AB_954967, Abcam, United Kingdom) and FITC-conjugated anti-rat (ab6730, RRID: AB_955327, Abcam, United Kingdom). Stained cells were fixed with 1% Para-Formaldehyde and stored at 4 °C until further analysis. Negative controls without primary antibodies were included. Flow cytometry was performed, and cell surface markers expression was acquired on a MACSQuant® Analyser 10 (Miltenyl Biotec, Germany) instrument, and the data were analysed using the FlowJo 10.4.2 (Flowjo Cloud, USA) software.

Furthermore, BMC cultured in tissue culture chamber (Sarsted, Mümbrecht, Germany) and fixed with paraformaldehyde 4% were characterised by immunostaining for CD90, CD45, CD44, CD29, CD13, CD68 according to the procedure described for flow cytometry. Cells were counterstained with Hoechst (1:200 dilution; Sigma-Aldrich, Switzerland) and examined under a high-resolution bright-field light microscope Nikon Eclipse Ni (Nikon, Japan), captured using a Nikon Digital Sight DS – Ri 1 camera and analysed by Nikon NIS elements software (Nikon, Japan).

Figure S3: Comparison of the expression profiles of macrophages cultured in F-BMC, BMC or fibrin conditioned medium.

Fold changes (FC) in gene expressions of pro-and anti-inflammatory markers were calculated for educated macrophages ($M_{(-)}$, $M_{(LPS,\ IFN)}$ or $M_{(Il4)}$) relative to uneducated ones. The values shown are mean ± SD. All n= 3 biologically independent samples constituted of macrophages pools, each pool was obtained from 3 animals, therefore, 9 animals per group. Statistics were performed using two-way ANOVA and a turkey's multiple comparison test as a post hoc test to compare a single effect for each macrophage subset and each gene.
For $M_{(IL4)}$, the downregulation of pro-inflammatory and upregulation of anti-inflammatory genes were similar when educated with F-BMC, BMC or fibrin except for *Cd163*, *Nos2 and Mcp1.*
For $M_{(-)}$ and $M_{(LPS,\ IFN)}$, F-BMC induced significantly pronounced changes of *Arg1*, *Il10*, Cd80, *Tnfa* compared to fibrin. When compared to BMC, the significative changes are macrophage subset dependent. Significant differences were measured in $M_{(-)}$ for *TGFb*, *Il10*, *IL6* and *TNFa.* For $M_{(LPS,\ IFN),\ the}$ most pronounced effects were observed for *Arg1* and *Tnfa.* Notably, fibrin induced downregulation of pro-and anti-inflammatory genes principally.

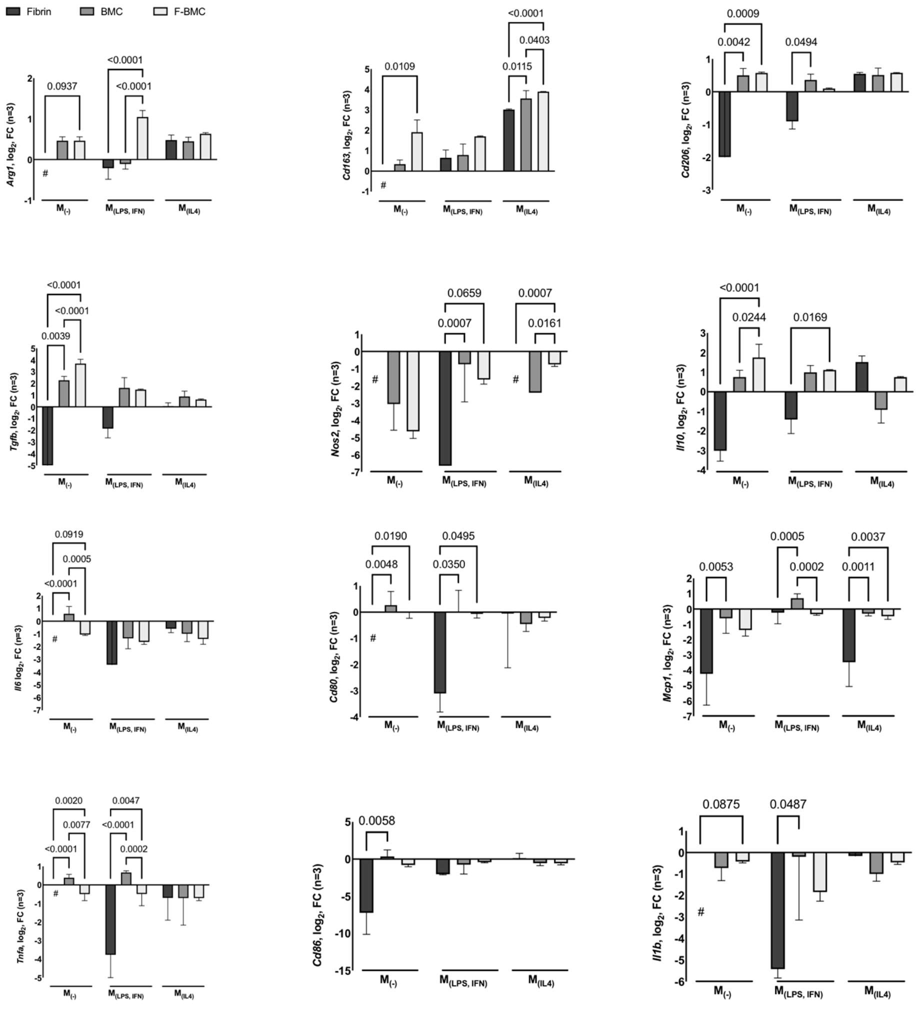

Table S2: Primers used for real-time PCR analysis

**Gene**	**Forward Primer (5'-3')**	**Reverse Primer (5'-3')**
Arg 1	TTGATGTTGATGGACTGGAC	TCTCTGGCTTATGATTACCTTC
Arg2	CGT CTC CCG TCT CCT CCA C	ACC ACC TCA GCC AGT TCC TG
b-Actin	AGCGTGGCTACAGCTTCACC	AAGTCTAGGGCAACATAGCACAGC
Cd163	TCAGCGTCTCTGCTGTCACTCA	CGTTCATGCTCCAGCCGTTA
Cd206	TGTGAGCAACCACTGGGTTA	GTGCATGTTTGGTTTGCATC
Cd80	TCGTACGTGGTGAAACACCTGA	CCGGAAGCAAAGCAGGTAATC
Cd86	GACACCCACGGGATCAATTA	AGGTTTCGGGTATCCTTGCT
Cxcl10	TTC CGT AAG CTA TGT GCA GGT A	TCA GGT GAA CTC AGA ACT GAT G
Gapdh	TGCCCCCATGTTTGTGATG	GCTGACAATCTTGAGGGAGTTGT
Gm-csf	GCA GAC CCG CCT GAA GCT AT	CGG CTT CCA GCA GTC AAA AGG
Icam1	TGA GCG ACA TTG GGG AAG AC	TCG CTC TGG GAA CGA ATA CAC
IFN gamma	TCG CAC CTG ATC ACT AAC TTC TTC	CGA CTC CTT TTC CGC TTC C
Il-1 beta	GCACCTTCTTTTCCTTCATCTTTG	TTTGTCGTTGCTTGTCTCTCCTT
Il-10	CTGTCATCGATTTCTCCCCTGT	CAGTAGATGCCGGGTGGTTC
IL-1a	AAG ACA AGC CTG TGT TGC TGA AGG	TCC CAG AAG AAA ATG AGG TCG GTC
IL-1ra	TCTGCAGGGGACCTTACAGT	GGTCTTCCTGGAAGTAGAAC
Il-4	ACC TTG CTG TCA CCC TGT TCT	AGC TCG TTC TCC GTG GTG T
Il-6	AAATGGTCCCCGGAGGT	AAGACACAGAGAGAAGCAATCCAAAC
iNos	ATGGAACAGTATAAGGCAAAC	GTTTCTGGTCGATGTCATGAG
M-csf	GGG AAT GGA CAC CTA CAG ATT TTG	AAA TTT ATA TTC GAT CAG GCA TGC A
Mcp-1	CTA TGC AGG TCT GTC ACG CTT C	CAG CCG ACT CAT TGG GAT CA
Mmp3	TCT TTC ACT CAG CCA ATG CT	GGG AGG TCC ATA GAG GGA TT
Mmp9	AGC CGG GAA CGT ATC TGG A	TGG AAA CTC ACA CGC CAG AAG
Rantes	CGTGAAGGAGTATTTTTACACCAGC	CTTGAACCCACTTCTTCTCTGGG
TGF beta	CTTCAGCTCCACAGAGAACTGC	CACGATCATGTTGGACAACTGCTCC
Timp1	CAG CAA AAG GCC TTC GTA A	TGG CTG AAC AGG GAA ACA CT
TNF alfa	TGCCTCAGCCTCTTCTCATTC	GCTCCTCTGCTTGGTGGTTT
Vcam1	ACA AAA CGC TCG CTC AGA TT	GTC CAT GGT CAG AAC GGA CT
